# Platelet formation and activation are influenced by neuronal guidance proteins

**DOI:** 10.3389/fimmu.2023.1206906

**Published:** 2023-06-15

**Authors:** Linyan Tang, Chao Liu, Peter Rosenberger

**Affiliations:** Department of Anesthesiology and Intensive Care Medicine; Eberhard Karls University Tübingen, Tübingen, Germany

**Keywords:** platelet, neuronal guidance protein, immunomodulation, inflammation, thrombosis

## Abstract

Platelets are anucleate blood cells derived from megakaryocytes. They link the fundamental functions of hemostasis, inflammation and host defense. They undergo intracellular calcium flux, negatively charged phospholipid translocation, granule release and shape change to adhere to collagen, fibrin and each other, forming aggregates, which are key to several of their functions. In all these dynamic processes, the cytoskeleton plays a crucial role. Neuronal guidance proteins (NGPs) form attractive and repulsive signals to drive neuronal axon navigation and thus refine neuronal circuits. By binding to their target receptors, NGPs rearrange the cytoskeleton to mediate neuron motility. In recent decades, evidence has indicated that NGPs perform important immunomodulatory functions and influence platelet function. In this review, we highlight the roles of NGPs in platelet formation and activation.

## 1 Introduction

Platelets are blood cells with fundamental functions. Platelets have been recognized as hemostasis-maintaining cells that sense vascular injury, adhere to collagen and each other to aggregate, and thus form thrombi to stop bleeding. Moreover, their functions in inflammation, cancer, and other physiological and pathophysiological processes have been described recently (1–3). During inflammatory responses, platelets directly interact with immune cells, including neutrophils (4–7), lymphocytes (8), monocytes (9–12), and macrophages (13–19), to mediate the activation, polarization, transmigration, and cytokine secretion of these cells. The interaction between platelets and the immune system has been established, and several specific terms have been applied to their intensive interactions. For instance, Stoll G. and Nieswandt B. coined the term ‘thrombo-inflammation’ to indicate that T-cell and platelet interactions occur during ischemia–reperfusion injury in stroke (8). Engelmann B. and Massberg S. introduced the term ‘immunothrombosis’ to describe the critical function of thrombosis in innate immunity. Immunothrombosis involves local platelets, fibrin, neutrophils, and monocytes, which interact and contribute to pathogen recognition and suppression (20). These ideas have been thoroughly reviewed (20–22).

The challenging roles played by platelets are possible because of the high reactivity of platelets to different molecules and stimuli and the precise intracellular and extracellular control of their responses and activities. Without sufficient regulatory structure, dysregulated hemostasis or excessive thrombosis can cause a range of fatal diseases, from hemophilia and von Willebrand disease to stroke, deep vein thrombosis, and pulmonary embolism.

Neuronal guidance proteins (NGPs) were originally identified by their attraction and repulsion functions, which promote synapse formation in the nervous system (23–26). In recent decades, an increasing number of studies have demonstrated the functions of NGPs in regulating basic immune functions, inflammation, oncology and platelet activation (2, 27–31). In this review, we summarize current knowledge about the modulatory functions of NGPs in platelet formation and activation.

## 2 Platelets

### 2.1 Platelet formation

Platelets are discoid anucleate cells generated from megakaryocyte (MK) cytoplasm. Hematopoietic stem cells (HSCs) exposed to thrombopoietin (TPO) differentiate into MKs (32, 33). MKs undergo polyploidization through DNA replication without cell division, accumulating from 2n to 64n and even 128n DNA pairs in a multilobe nucleus, a process named endomitosis (34, 35). The formation of an invaginated membrane system (IMS) is another characteristic of MK maturation, and the process is well established (36). Membrane assembly starts at the cell periphery and is positioned precisely between nuclear lobes. The amount of invaginated membrane and extent of nuclear lobulation are correlated, and there is a close association between cleavage furrow formation and inhibited cytokinesis during the formation of the IMS (36). During the process, Golgi complexes and the endoplasmic reticulum (ER) are in close contact with the IMS, suggesting the mechanism by which membranes are formed and lipids are transferred (36).

After maturation *via* polyploidization and IMS formation, cytoplasmic branches called proplatelets protrude from MKs. Proplatelets are elongated MK protrusions that extend into sinusoidal microvessels in the bone marrow and shed platelets from the tips of the protrusion branches (37). This process was clearly demonstrated *in vitro* by J. E. Italiano et al. (37). Mature MKs spread and form large pseudopodia on the polarized side opposite the side with polyploid nuclei. The pseudopodia extend and bend dynamically and form new branches into bending sites until the whole cytoplasm transforms into proplatelet tubes, which may undergo anastomosis with each other. Proplatelets contract discontinuously along the long axis to produce areas of swelling into ‘beads’. Subsequently, proplatelet tips adhere and extend to form flat lamellipodia, and during this process, the ends of a proplatelet crawl away from the cell center (37). The process of proplatelet formation and platelet release *in vivo (* 38) is slightly different from that *in vitro*, where sinusoidal vessel walls, blood flow shear stress in microvessels and the microenvironment in bone marrow are absent. With multiphoton intravital microscopy, T. Junt et al. demonstrated that MKs are in close proximity to bone marrow sinusoids and are relatively stationary compared to resident bone marrow cells (39). MKs form protrusions into bone marrow, and these protrusions extend faster *in vivo* than they do *in vitro* (3.9 µm/min *vs*. 0.85 µm/min) (39). Proplatelets extend through the sinusoidal wall into the microvessel lumen in the bone marrow and are cleaved and released into blood circulation. In contrast to an *in vitro* model, in which all of the MK cytoplasm can be observed to transform into proplatelet nets within 4 hours (37), the proplatelets extended *in vivo* consist of ~6% of the average volume of an MK, and proplatelets are released approximately every 7 hours (39). This variation indicates that *in vivo*, proplatelet protrusion and elongation is a gradual process that is effectively controlled. Released proplatelets are easily recognized in the peripheral blood circulation, providing further evidence for the theory that platelets are ultimately formed in peripheral circulatory structures, such as pulmonary arterioles (40, 41), and blood flow shear stress plays critical roles in this process (38).

### 2.2 Role of the cytoskeleton in platelet formation

**Figure 1:**
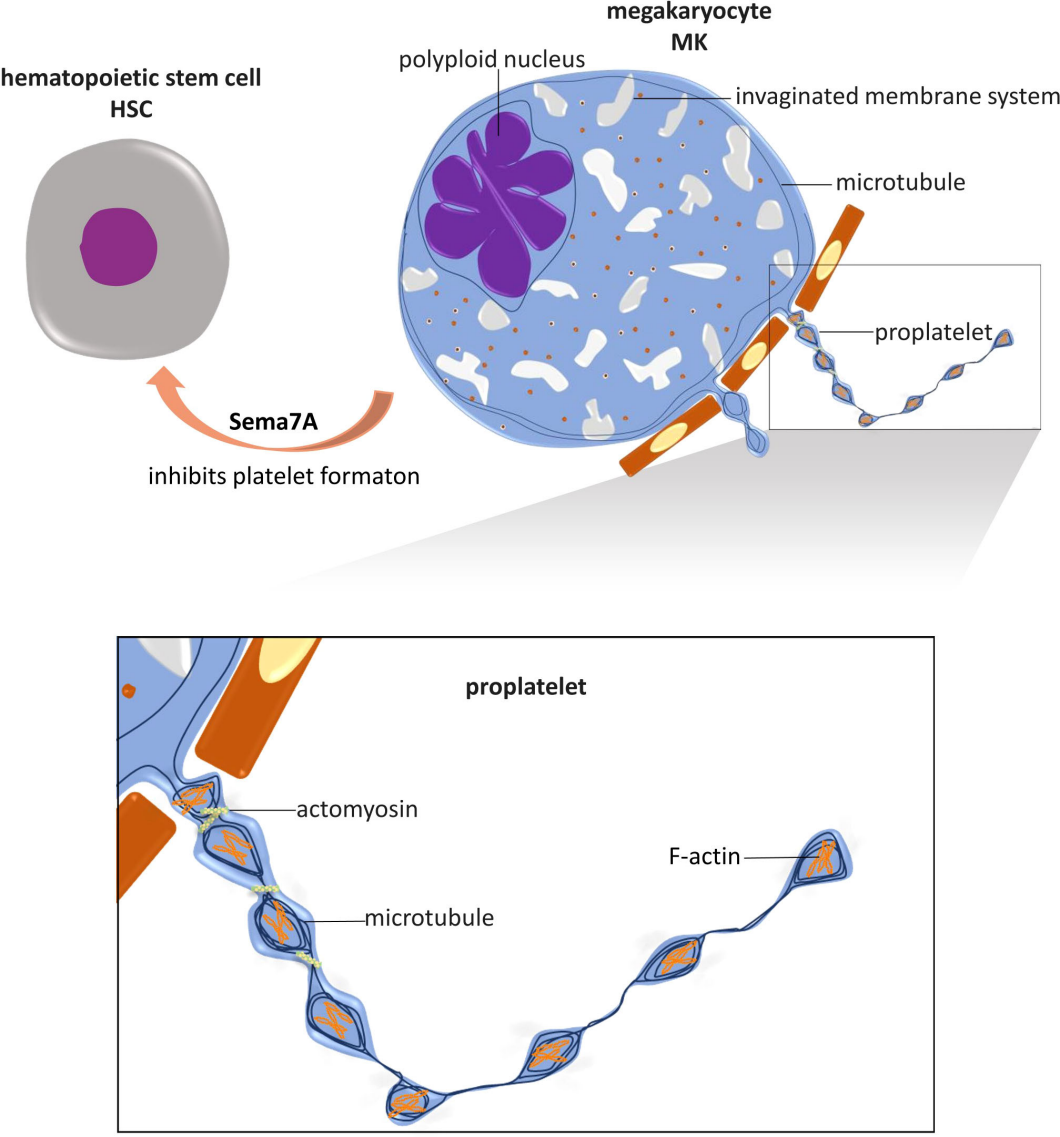
Role of NGP and cytoskeleton in platelet formation. Megakaryocytes (MKs) are differentiated from hematopoietic stem cells (HSCs), and their maturation is marked by the polyploidized nucleus and the formation of an invaginated membrane system. Mature MKs extend cytoplasmic branches, named proplatelets, into sinusoidal microvessels in the bone marrow and shed platelets. In this process, microtubules are fundamental for proplatelet protrusion and elongation. Actin polymerization plays an important role in the dynamic bending and branching of proplatelets. Actomyosin provides mechanical force for proplatelet segmental contraction and intermediate narrowing, by which the size of platelets is limited. The NGP known as Sema7A has been shown to regulate platelet formation by inhibiting MKs differentiation. This is the only known NGP with this capability.

In the dynamic morphogenesis of proplatelets and final platelet formation, the intracellular cytoskeletal system in MKs, comprising actin, myosin, and microtubules, plays crucial roles (**Figure 1**).

When actin filament polymerization is inhibited by cytochalasin B (42), the bending dynamics of MK protrusions and branching of proplatelets are significantly decreased (37). Indicating the importance of F-actin in platelet formation, platelet-like swelling areas in a proplatelet consist of a meshwork of densely packed F-actin (37).

In the presence of cytochalasin B, MKs produce proplatelet extension without swollen bead formation (37). This outcome is unsurprising since cytochalasin B reduces actomyosin viscosity and contractile microfilament formation (43), inhibiting segmental contraction and intermediate contractile narrowing in the proplatelets. When myosin-9, also called nonmuscle myosin heavy chain-IIa (NMMHC-IIA), is rendered defective by mutation of the gene that encodes it, MYH9, myosin filament formation is disrupted (44), and contractile function is inhibited (45); therefore, MKs produce giant platelets (46–50). For example, these large platelets are characteristic of May–Hegglin anomaly (47), Fechtner syndrome, Sebastian syndrome (46, 48), and Epstein syndrome (49), which are considered MYH9-related genetic diseases (49, 50).

The nonmuscle myosin II molecule is a hexamer composed of two 230 kDa heavy chains, two essential light chains (ELCs) of 17 kDa, and two regulatory light chains (RLCs) of 20 kDa (51). The regulation of nonmuscle myosin II relies on the phosphorylation of serine 19 and threonine 18 on RLCs, which is mediated by different kinases, but in MKs and platelets, the most important players are Rho-GTPases, especially RhoA and CDC42 (52, 53). Tissue-specific gene knockout of RhoA in MKs led to macrothrombocytopenia. RhoA^-/-^ MKs produce 50% fewer platelets and exhibit a 25% increase in total platelet volume, with the large platelets formed in RhoA^-/-^ MKs being rounder than those in wild-type MKs (54). RhoA regulates proplatelet formation by inhibiting cytoplasmic protrusion extension (55–57). *In vitro*, retroviral overexpression of RhoA leads to reduced MK formation, but MKs transfected with retroviruses encoding dominant-negative RhoA produce more proplatelets (56). Inhibition of RhoA and its main downstream effector ROCK leads to reduced phosphorylation of RLCs in NM II and increased proplatelet formation (56). In contrast to the ‘STOP’ signal function of RhoA, CDC42 seems to exert a ‘GO’ signal function, driving MK proplatelet formation. Inhibition of CDC42 expression significantly reduces proplatelet formation, while MKs overexpressing CDC42 produce markedly more proplatelet protrusions (58).

Electron micrographs have clearly revealed the parallel bundles of structural microtubule skeletons in proplatelets, and increasing evidence indicates a fundamental function for microtubules in proplatelet protrusion and elongation (37, 39, 59, 60). Stabilizing microtubules with Taxol significantly decreases the number and length of proplatelet protrusions from MKs and leads to fewer and shorter but thicker protrusions^18^. Moreover, microtubules disrupted by nocodazole (39), vincristine sulfate or colchicine show highly suppressed proplatelet formation and elongation (59). A β1-tubulin gene (TUBB1) mutation in humans results in the formation of abnormally large proplatelets and macrothrombocytopenia (60). Mice lacking the transcription factor NF-E2, which inhibits the transcription of β1-tubulin, a main tubulin isoform in MKs, exhibit severe thrombocytopenia. MKs with NF-E2 knocked out undergo polyploidy, and invaginated membranes accumulate but never form proplatelets (61).

### 2.3 Platelet activation

At the intracellular and molecular levels, platelet activation involves intracellular calcium flux, negatively charged phospholipid translocation, granule release, and shape change.

Platelet calcium flux is stimulated by agonists mainly through G protein-coupled receptors (GPCRs) or ITAM-linked receptors (ILRs) (62, 63). Receptors for thrombin, ADP and thromboxane A2 (TxA2) are GPCRs and signal through phospholipase (PLC) β. ILRs include GPVI and C-type lectin-like receptor 2 (CLEC-2), which regulate PLCγ isoforms (62). Activation of both pathways generates inositol 1,4,5 trisphosphate (IP3), which binds to the inositol phosphate-sensitive (IPS) receptor on dense tubules to promote Ca^2+^ release and increase the cytoplasmic Ca^2+^ concentration. Subsequently, the increase in Ca^2+^ level activates the cytoskeletal system and regulates various cell processes, such as phospholipid translocation; granule release; cell shape change; and protein trafficking, redistribution and activation (1). Negatively charged phospholipids translocate from the inner leaflet to the outer membrane surface in activated platelets. The negatively charged platelet surface facilitates coagulation by enabling platelet binding to the coagulation enzyme complex, which activates serine proteases and subsequently activates thrombin (64). Activated platelets release α-granules, dense granules, and lysosomes. These components play various roles in regulating physical processes, including hemostasis and coagulation, inflammation, vasoconstriction, and angiogenesis (56, 65–71). Notably, some of these components, such as vWF, fibrinogen, and growth factors, are released into the extracellular microenvironment. Other components, such as integrin α_IIb_β_3_, GPVI, components of the GPIb-IX-V complex and P-selectin, are fused or redistributed into the cytoplasmic membrane, where they play critical roles in signal transduction. Activated platelets transform from a regularly round discoid shape to an irregular shape, forming actin-enriched sheets in lamellipodia and numerous extended filopodia that facilitate platelet aggregation and adhesion (1).

Activated platelets fulfill their function in hemostasis, thrombosis and inflammation *via* adhesion (to collagen or other types of cells) and aggregation (to other platelets). In injured vessels, collagen in the subendothelial matrix is exposed and binds to two prominent receptors on platelets, GPVI and GPIa/IIa (integrin α_2_β_1_). Endothelial cells undergoing injury or inflammatory responses release von Willebrand factor (vWF) from Weibel-Palade bodies (WPBs), which bind to the platelet GPIb/V/IX complex (1, 72). These binding events initiate the activation of a signaling cascade, leading to Ca^2+^ release and the subsequent activation processes in platelets and ultimately to GPIIb/IIIa (integrin α_IIb_β_3_) activation (1, 73). Activated platelets redistribute more GPIIb/IIIa onto the cell surface by granule secretion, and more importantly, these receptors are activated, showing high affinity for their ligand fibrinogen (74). One fibrinogen binds two molecules of GPIIb/IIIa, promoting stable platelet aggregates (75).

### 2.4 Platelet activation and cytoskeleton

From the information presented thus far, it is clear that platelet activation is a profoundly dynamic and orchestrated process. Therefore, it is not surprising that the cytoskeletal system exhibits fundamental functions in this process (1, 76–78). Actin polymerization is not only critical for filopodia formation and extension (1) but also important for α-granule secretion (76). In activated platelets, peripheral microtubule coils expand due to the increased Ca^2+^ concentration and fold into the cell center. This change promotes platelet transformation from a discoid to a spherical shape (78).

The platelet cytoskeleton is regulated by the Rho GTPase family members RhoA (54, 79–82), Rac1 and Cdc42, which collectively mediate platelet activation. Specifically, RhoA is essential for platelet shape change, α-granule and dense granule secretion, integrin α_IIb_β_3_ activation, integrin-mediated clot retraction, and stable thrombus formation (54). Rac1 deficiency blocks granule secretion, lamellipodia formation and platelet aggregation (80, 81). Cdc42 and actin polymerization are critical for integrin α_2_β_1_ (also known as GPIa/IIa) activation, which induces the tight attachment of platelets to collagen (82).

### 2.5 Regulation of platelet activation

In the blood, platelet activation needs to be quick and effective to limit blood loss and restore blood vessel integrity. Simultaneously, platelet activation requires tight control to limit the scale of thrombosis and to maintain vascular patency and blood supply to tissues. Therefore, platelet functions must be both positively and negatively regulated to maintain a balance of effective reactions and controlled scale.

Positive drivers of platelet activation include thrombin, adenosine diphosphate (ADP), TxA2, and epinephrine. All of these agonists cooperate with one or more GPCRs and subsequently elevate the Ca^2+^ concentration and facilitate various platelet activation processes (83, 84).

The negative regulators of platelet activation maintain platelet quiescence in the blood circulation and control the degree of thrombosis. These inhibitory factors are generated in endothelial cells (nitric oxide, NO and prostacyclin PGI_2_), on the platelet surface (platelet endothelial cell adhesion molecule-1, PECAM-1), or in platelets (85). Knowledge obtained to date on these regulators has been effectively summarized elsewhere (85, 86).

Another emerging group of platelet functional regulators comprises neuronal guidance proteins (NGPs). Our understanding of these factors is still in its infancy, but increasing evidence indicates their promising prospects.

## 3 Neuronal guidance proteins influence platelet formation and activation by regulating the cytoskeletal system

### 3.1 Neuronal guidance proteins

Neuronal guidance proteins (NGPs), also named axon guidance proteins, are various proteins that guide neurons in nervous system development both during embryogenesis and in neonates (87, 88). NGPs are composed of (but not limited to) netrin and its receptor called deleted in colorectal cancer (DCC); slit and its receptor Robo; Ephs and their receptors called ephrins; RGM and its receptor neogenin; Wnt and the receptor Frizzled; and protocadherins (Pcdhs) and semaphorins and their receptors called plexins (23, 87, 88). Many NGPs are evolutionarily conserved (89). NGPs engage in overlapping attraction or repulsion signaling with billions of neurons to induce the assembly or collapse of growth cones (23), suppress or promote the growth of axons and dendrites (24), modulate synaptic contacts (25) and prune axons (26) to refine neuronal circuits. During this complicated process, some NGPs provide long-distance chemoattractive or chemorepulsive signals to guide neuron axons; for example, netrin-DCC and Slit-Robo navigate neurons during spinal cord development (87, 90). Some NGPs serve as surrounding repulsive signals to inhibit inappropriate synaptic contacts, such as that between semaphorins and plexins (87, 91), and Pcdhs generate self-avoidance signaling that inhibits neurons from inducing nonfunctional synapse formation (87, 92). Although various and redundant signaling proteins are involved, the effectors for all NGPs are consistent and unique and originate in the cytoskeletal system. By binding to their receptors, NGPs regulate the action of small GTPases and subsequently influence cytoskeleton rearrangement and neuron motility (87, 88, 93). Given the fundamental function of the cytoskeletal system in platelet formation and activation, it is not surprising that NGPs play critical roles in regulating platelet activity.

Increasing evidence has proven that NGPs are involved in many physiological and pathological processes, such as immune reactions (29), tumor invasion and metastasis (94, 95), and tissue repair and regeneration (96, 97). In this review, we provide up-to-date knowledge about their regulatory functions in platelet formation and activation. All NGPs involved in platelet formation and activation are shown in (**Table 1**).

**Table 1:** NGP involvement in platelet development and activation.

Ligands*	Receptors*	Function	Molecular mechanism	Refs
Sema3A	neuropilin-1 PlexinA1	downregulates α-granule and dense granule secretion, inhibits integrin α_IIb_β_3_ activation, suppresses platelet adhesion and aggregation	Rac-1 cofilin actin	(98)
Sema7A	GPIb	upregulates granule secretion and P-selectin and integrin α_IIb_β_3_ distribution, enhances platelet aggregation.		(2)
	β_1_ integrin	inhibits platelet formation.		(99)
Sema4D	PlexinB1 CD72	promotes platelet activation and aggregation	Syk Ca^2+^	(100)
	PlexinB2	downregulates P-selectin expression, inhibits platelet adhesion to fibrinogen	targeted by miRNA-126-3p	(101)
EphA4 EphB1	ephrinB1	induces α-granule secretion, promotes platelet adhesion and aggregation	Rap1B myosin-integrin β_3_ binding	(102, 103)
Slit2	Robo-1	inhibits platelet spreading, adhesion and granule secretion; inhibits thrombus formation	Akt	(104)

*The role as ligand or receptor for some NGPs is alternative. For instance, Sema4D usually functions as a ligand for PlexinB2, but some research has also proven that Sema4D works as a receptor, while PlexinB2 functions as a ligand (105).

### 3.2 Semaphorin 3A inhibits platelet activation

Semaphorin 3A (Sema3A) is a secreted homodimer that functions through the receptor complex comprising neuropilin-1 and PlexinA1 (106). Sema3A binds to neuropilin-1, while PlexinA1 mediates intracellular signaling (107, 108). In the nervous system, Sema3A inhibits sympathetic neuron migration and modulates sympathetic neuron arrest and aggregation in the proper position (109). The genes for Sema3A, neuropilin-1 and PlexinA1 (PLXNA1) are all orthologous between humans and mice, and sequence alignment analysis with the Constraint-based Multiple Alignment Tool (COBALT) from the National Center for Biotechnology Information (NCBI) has shown a 100% match for all three protein-encoding genes (110). The functions of Sema3A in the immune system have been established: i) Sema3A inhibits T-lymphocyte activation, proliferation, and cytokine production (111–113); ii) Sema3A stimulates dendritic cell (DC) activation and plays a chemorepellent role in DC migration; and iii) Sema3A regulates monocyte and macrophage migration and polarization.

Western blotting and RT−PCR have demonstrated that the Sema3A receptors neuropilin-1 and PlexinA1 are abundantly expressed on human platelets (98). Sema3A inhibits GPIIb/IIIa activation on human platelets and subsequent platelet aggregation (98). Sema3A has also been proven to downregulate α-granule and dense granule secretion of human platelets (98). Moreover, it suppresses human platelet adhesion and spreading on fibrinogen-coated and uncoated surfaces, indicating that this inhibitory function is either GPIIb/IIIa-dependent or GPIIb/IIIa-independent (98). All of these inhibitory functions can be explained by the significant inhibition mediated by Sema3A on Rac-1 activation in stimulated human platelets, which decreases cofilin phosphorylation and inhibits actin polymerization (98). In addition, the inhibitory function of Sema3A does not depend on either intracellular Ca^2+^ concentrations or changes in cAMP or cGMP levels in activated platelets (98).

The inhibitory effect of Sema3A on platelet activation is consistent with some clinical research (114). Guo Q et al. showed that in certain autoimmune diseases, such as systemic lupus erythematosus (SLE), the serum concentration of Sema3A is significantly lower than that in serum from healthy individuals. In SLE patients with thrombocytopenia, the Sema3A concentration in serum is even lower than that in uncomplicated SLE cases and is highly correlated with the platelet count (114).

#### 3.3 Semaphorin 7A regulates platelet formation and activation

Semaphorin 7A (Sema7A, also named CD108) is a glycosylphosphatidylinositol (GPI)-anchored membrane protein that functions through its receptors PlexinC1 (also known as CD232) (115), β_1_ integrin (also known as CD29) (116) and platelet GPIb (2). The SEMA7A, PLXNC1 and ITGB1 (β1 integrin) genes are orthologous between humans and mice and are conserved in humans, rhesus monkeys, mice, rats, chickens, zebrafish, and frogs (110). In the nervous system, Sema7A promotes axon outgrowth and regulates axon tract formation, and this function depends on Sema7A binding to integrin β_1_ but not to PlexinC1 (116). In the immune system, Sema7A interacts with PlexinC1 to promote monocyte activation (115, 117). Sema7A also promotes neutrophil extravasation in hypoxia-induced inflammation (118). Sema7A is also expressed on activated T lymphocytes and stimulates monocytes and macrophages to produce cytokines by binding to integrin α_1_β_1_ (also named very late antigen-1, VLA-1) (119).

Our research group found that Sema7A increased platelet activation in myocardial ischemia−reperfusion injury (MIRI) (2). After MIRI model mice were injected with recombinant mouse Sema7A (rmSema7A), platelet granule secretion was elevated, and P-selectin distribution on the cytoplasmic membrane was increased. Platelet aggregation was also enhanced with additional activated integrin α_IIb_β_3_ molecules on the cell surface. Moreover, Sema7A knockout or antibody blockade led to reduced platelet activation in MIRI mouse models, as proven by the diminished expression of P-selectin and integrin α_IIb_β_3 on_ the platelet surface (2). Knocking out Sema7A decreased platelet neutrophil complex (PNC) formation, decreased the neutrophil transmigration rate into injured myocardial tissues, and markedly reduced the infarct area in the mouse model of MIRI (2).

However, Sema7A alone did not induce resting platelet activation or aggregation under static conditions but facilitated shear stress-activated platelet adhesion and thrombus formation by increasing P-selectin secretion onto the platelet surface and by activating integrin α_IIb_β_3_ (2). This explanation is reasonable since Sema7A has been proven to promote intracellular actin polymerization and cytoskeletal rearrangement (120). Interestingly, this function of Sema7A depends on GPIb on platelets; when we blocked GPIb with p0p/B (121), the function of Sema7A was no longer detected (2).

In contrast to the promoting effect of Sema7A on platelet activation, Sema7A has been proven to inhibit platelet formation from MKs (99) **(** **Figure 1** **)**. Hematopoietic CD34+ progenitor cells differentiate into all blood cell lines, including MKs and platelets (122). *In vitro* Sema7A exposure reduces hematopoietic stem cell (CD34+) differentiation into MKs and decreases the platelet formation rate, and these functions depend on Sema7A binding to its receptor β_1_ integrin (99). Sema7A facilitates hematopoietic progenitor cell differentiation into CD14+ cells (99) (monocytes (123)) and induces MKs and platelets to produce increased levels of proinflammatory cytokines, including IL-6, IL-8, and granulocyte-macrophage colony-stimulating factor GM-CSF (99). In chemotherapy patients, Sema7A expression is upregulated both on the surface of peripheral blood mononuclear cells (PBMCs) and in serum (99). The proinflammatory and inhibitory effects of Sema7A on MK differentiation and platelet formation together may lead to thrombocytopenia in chemotherapy patients (99).

### 3.4 Semaphorin 4D enhances platelet reaction

Semaphorin 4D (Sema4D, also named CD100) is a transmembrane protein expressed on various cell types, including platelets, neutrophils, T cells, B cells, monocytes and dendritic cells (DCs), in the immune system and can be found in the lungs, brain, kidneys, heart, and spleen (105, 124–127). It was discovered on human T lymphocytes and named CD100 (128) in 1992, and its discovery was the primary evidence for Semaphorin expression in the immune system (125). On the cell membrane, Sema4D forms homodimers with monomers linked by disulfide bridges (129). The extracellular region of Sema4D is cleaved, releasing a soluble form, and this exodomain cleavage is mediated by the metalloprotease ADAM17 (130). Sema4D binds three receptors, PlexinB1, PlexinB2 and CD72 (105, 131), and its binding affinity for these receptors seems to vary depending on the cell type with the expressed receptors and the cell condition (131). The SEMA4D, PLXNB1 and PLXNB2 genes are orthologous between humans and mice and are conserved in humans, rhesus monkeys, mice, rats, chickens and zebrafish (110).

Research has proven that Sema4D and its receptors play critical roles in the immune system. For example, Ponnat I. et al. proved that Sema4D binds to PlexinB1 on monocytes and DCs to influence the immune cell migration process (27). A Sema4D-knockout mouse model of foreign antigen-induced crescentic glomerulonephritis has been found to recruit fewer macrophages to the glomeruli and exhibit fewer activated T and B cells in lymph nodes than wild-type mice (132). Nishide M. et al. (105) illustrated that the soluble Sema4D concentration was increased in patients presenting with antineutrophil cytoplasmic antibody (ANCA)-associated vasculitis (AAV) and that this increase was accompanied by decreased expression of Sema4D on the neutrophil surface. Soluble Sema4D facilitated endothelial cell inflammation, while PlexinB2 on endothelial cells bound to membrane Sema4D on neutrophils and inhibited neutrophil extracellular trap (NET) formation.

The role of Sema4D in regulating platelet activation has been demonstrated mainly by the research group of Lawrence F. Brass (100, 133, 134), who proved with western blots and flow cytometry that human platelets expressed Sema4D and that the expression increased 2-fold after PMA stimulation, after which total cleavage occurred that was mediated through ADAM17 action (100). This cleavage seemed to depend on and follow platelet aggregation since blocking the binding of fibrinogen to integrin α_IIb_β_3_ inhibited Sema4D cleavage (100). Platelets also express the receptors of Sema4D with CD72 in human platelets and with PlexinB1 in both human and mouse platelets. Immunoblotting confirmed that the expression of CD72 on human platelets was significantly upregulated by PMA stimulation (100). Platelets from Sema4D-knockout mice showed impaired aggregation *in vitro*, while coagulation and thrombus formation after vascular injury were inhibited *in vivo (* 100). Brass et al. concluded that platelet membrane Sema4D promoted platelet activation and aggregation by binding CD72 or PlexinB1 on adjacent platelets (100).

Subsequent research from the same group revealed the mechanism for impaired collagen-induced platelet aggregation in Sema4D-knockout mice. They found that Sema4D was crucial for splenic tyrosine kinase (Syk) activation in collagen-stimulated platelets (133). Knocking out Sema4D in mice suppressed the activation of Syk, which subsequently caused lower levels of Ca^2+^ to be released after collagen-induced platelet activation (133). The important role played by Syk in regulating the cytoskeletal system has been shown by other studies (135–137). In thrombin-stimulated platelets, Syk is relocated to the actin filament network and promotes actin polymerization (136). Syk also regulates microtubules by binding and phosphorylating β-tubulin and α-tubulin (135, 137).

Moreover, the function of Sema4D in dyslipidemia-induced atherosclerosis has been described (134). Platelet activation plays important roles in promoting atherosclerosis in dyslipidemia (134, 138). Both native and oxidized low-density lipoprotein (LDL) lead to platelet hypersensitivity to agonists and increased aggressive adhesion, granule secretion and aggregation, which increases the risk of athero-occlusion and death *via* cardiovascular disease (138). In mice with dyslipidemia, platelet accumulation in the injured endothelium is 3-fold greater than that in the endothelia of healthy mice (138). Sema4D knockout inhibits collagen-induced platelet accumulation and contact *in vitro*, and it leads to decreased platelet accumulation in the acutely injured endothelium in mice with normal lipid levels and those with dyslipidemia (134).

Cleavage of the Sema4D exodomain (130) seems to involve the same mechanism as that underlying the shedding of GPIbα (139), GPVI (140), and PECAM-1 from platelets (141). In resting platelets, calmodulin binds the Sema4D cytoplasmic domain Arg762-Lys779, whereas inhibition or deletion of calmodulin causes Sema4D cleavage without triggering platelet activation (with the P-selectin level as the measured marker) or ADAM17 reaction (130).

### 3.5 Plexin B2 suppresses platelet activation

Although platelets are anucleate cells and do not carry genomic DNA, increasing evidence has proven that platelets can respond to stimuli at the protein translational level because they contain abundant messenger RNA (mRNA); microRNA (miRNA), which functionally regulates mRNA transcription; and necessary organelles, such as the rough endoplasmic reticulum and ribosomes (142–145). Platelets express 32% of all human genes at the mRNA level (146, 147) and can synthesize various proteins, including the major membrane glycoproteins GPIb, GPIIb, and GPIIIa and granule proteins such as vWF and fibrinogen (148). In platelets, mRNA translation is regulated by miRNAs, which represent the majority of all small RNAs (~80%) (142).

The function of PlexinB2 in platelet formation and activation has been highlighted by research performed with miRNA-126-3p (101). In human platelets, the mRNA of PLXNB2 (the gene encoding the protein PlexinB2) has been confirmed to be a target of miR-126-3p. After MKs were transfected with miR-126-3p, the expression of PLXNB2 mRNA and protein was significantly downregulated (101). Compared to the mock cells, miR-126-3p-transfected human MKs expressed 30% more CD62P in thrombin-stimulated PLS (platelet-like structures) and exhibited 156 ± 14.9% greater adhesion to the fibrinogen-coated chamber (101). In line with this finding, silencing PlexinB2 in human MKs enhanced platelet adhesion to the fibrinogen-coated channel. These results indicate the inhibitory function of PlexinB2 in platelet activation.

However, as PlexinB2 is the main receptor of Sema4D, the inhibitory function of PlexinB2 appears to slightly contradict the facilitative effect of Sema4D on platelet responses.

### 3.6 Ephrins and Eph promote platelet activation mediated by Rap1B

Ephrins and Eph receptors belong to the receptor tyrosine kinase (RTK) superfamily, and ligand binding induces tyrosine phosphorylation of their cytoplasmic region (149). Eph receptors are composed of two classes, EphA (EphA1-EphA10) and EphB (EphB1-EphB6), which are distinguished and named according to extracellular domain sequence (149). The ligands of Eph receptors, ephrinA1-A5 and ephrinB1-B3, are membrane-binding proteins and are anchored to the cytoplasmic membrane *via* their GPI domain (ephrinA) and transmembrane region (ephrinB) (149). Membrane-bound ephrin binding induces Eph receptor phosphorylation, but soluble ephrin binding to Eph receptors does not trigger receptor c-terminal phosphorylation (149). During nervous system development, the Eph receptor density gradient on retinal ganglion cells is similar to the density gradient of ephrin expressed on subcortical neurons, which helps maintain proper neuronal axon projection *via* a ‘topographic mapping’ function (87). EPH and EPH-related receptors are evolutionarily conserved; for instance, the EPHA4 and EPHB1 genes are orthologous between humans and mice (110). These proteins have been proven to be important after an inflammatory response (150); for example, EphA2 and ephrin-A1 regulate endothelial permeability by increasing the Src kinase level and upregulating Rho-GTP expression, which subsequently leads to the opening of adherens junctions (151, 152). Additionally, Eph/ephrin plays roles in angiogenesis (153) and the response to spinal cord injury (154).

Human platelets have been proven to express the Eph kinases EphA4 and EphB1 and the ligand ephrinB1 by Lawrence F. Brass with western blots and fluorescence staining (102). With actin visualization by rhodamine-phalloidin, they proved that clustering of both EphA4 and ephrinB1 promoted human platelet adhesion and spreading on a fibrinogen-coated surface. In addition, α-granule secretion and P-selectin expression on human platelets were also induced by clustering of both EphA4 and ephrinB1, as demonstrated by flow cytometry. All these responses indicated cytoskeletal reorganization, although the platelet cytosolic Ca^2+^ concentration was not increased by EphA4 and ephrinB1 clustering (102). The interaction of EphA4 and ephrinB1 activated Rap1B, a member of the Ras superfamily, in human platelets (102, 103). Moreover, blocking the Eph/ephrin interaction inhibited human platelet aggregation, suggesting that the Eph/ephrin interaction plays a critical role in stabilizing platelet plugs (102, 103). In subsequent research, this research group demonstrated that blocking the Eph/ephrin interaction significantly inhibited platelet clot retraction, which is fundamental for thrombus stability (155). The basic mechanism is based on the Eph/ephrin interaction promoting integrin β_3_ binding to myosin, which provides the force needed for platelet clot retraction (155).

### 3.7 The Slit2 and Roundabout interaction inhibits platelet activation

Insects and vertebrates employ a symmetric bilateral nervous system in which the two sides are mirror images that are closely connected with contralateral commissural axons that cross the midline structure. During the development of this symmetric nervous system, commissural axons are guided across the midline by long- and short-range attractive and repulsive signals (156). The long-range signals are emitted by chemoattractants called netrins, and the short-range signals are emitted by the contact-mediated repellent Slits and Roundabout (Robo) (156). Slits and Robo are evolutionarily conserved (89, 156). For example, the Slit2 gene is conserved in humans, rats, mice, zebrafish and *C. elegans*, and an analysis with multiple sequence alignment (MSA) showed 100% matches between human and mouse Robo 1 (110). Robo receptors are immunoglobulin proteins that inhibit axons from crossing the midline. When axon growth cones express high Robo levels, they never cross the midline; in contrast, midline-crossing axons express high Robo receptor levels only after crossing the midline, not before. *Robo* gene mutation leads to axon crossing and recrossing multiple times. Subsequent research has identified slits as ligands of Robo receptors, and in slit-mutant embryos, axon growth cones cross the midline but do not migrate further (157).

Evidence has proven that slits and Robo are important for the development of non-nervous system organs, such as the lungs, kidneys and heart (158–161). An increasing number of studies have shown that slits and Robo play fundamental roles in inflammation. Slit2 has been proven to inhibit lymphocyte and neutrophil migration due to chemotaxis (162, 163), decrease leukocyte adhesion (164, 165), and suppress leukocyte transendothelial migration (164, 165).

The Robo-1 receptor has been shown to be distributed on the surfaces of both human and murine MKs and platelets by Patel S. with western blots, flow cytometry and immunofluorescence microscopy (104). Through the leucine-rich regions in its N-terminus, Slit2 directly binds Robo-1 (104). By binding to Robo-1, slit2 inhibits human platelet spreading on a fibrinogen-coated surface by inhibiting the formation of lamellar sheets between filopodia, platelet adhesion to immobilized collagen under fluid shear stress and nonstress conditions, and platelet granule secretion (104). Subsequently, slit2 inhibits thrombus formation in injured vasculature and prolongs the bleeding time in a murine tail bleeding model (104). These inhibitory effects are realized by the effect of slit2 on Akt activation in human platelets. Immunoblotting has proven that slit2 inhibits Akt phosphorylation in human platelets but exerts no effect on Rac1, Cdc42, extracellular signal-regulated kinase (ERK), or p38 mitogen-activated protein kinase (MAPK) activation (104).

**Figure 2:**
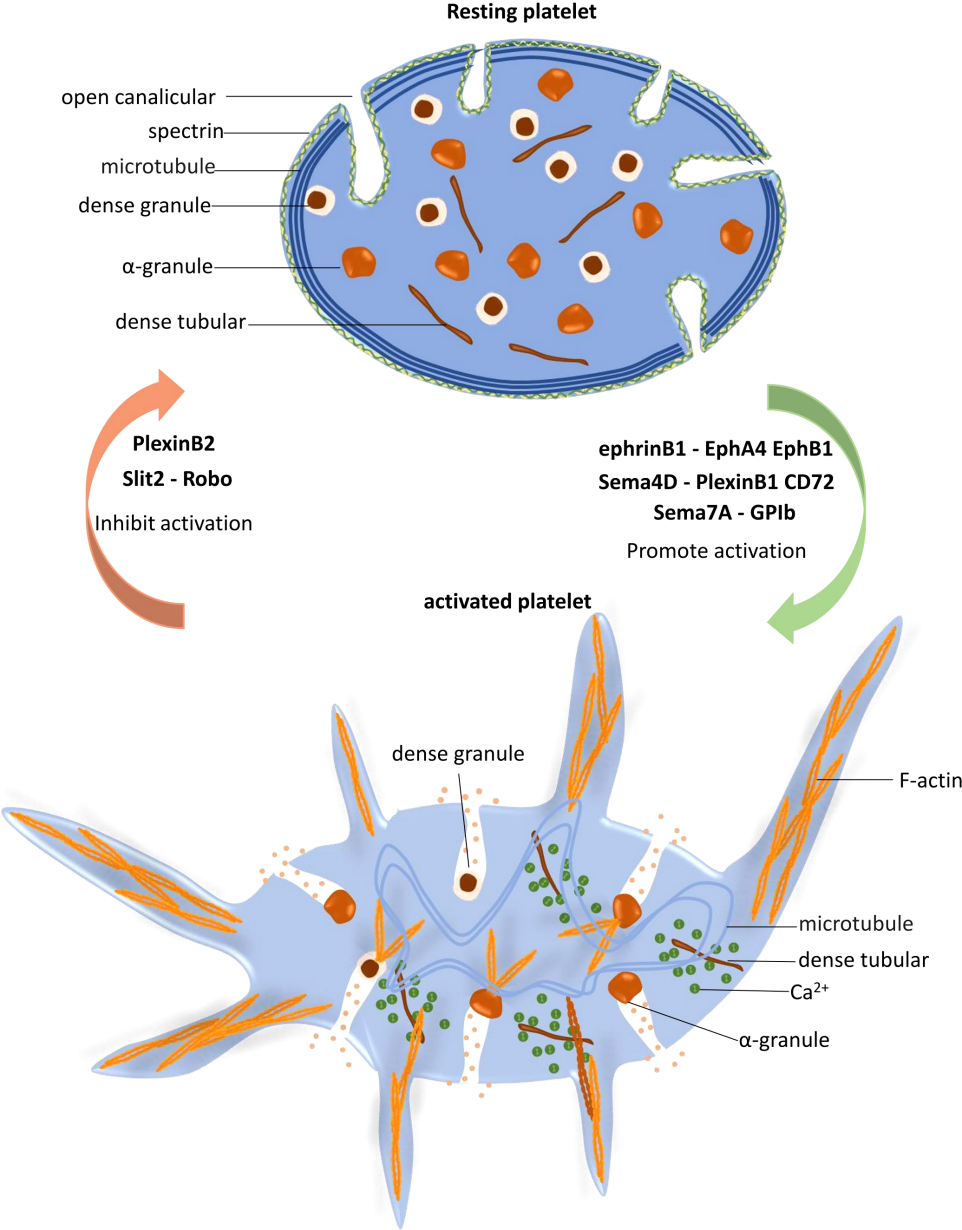
Platelet activation is regulated by neuronal guidance proteins (NGPs). In resting platelets, the spectrin-based skeleton supports the plasma membrane and the open canalicular system (OCS), and the marginal microtubule coils maintain the characteristic discoid shape of platelets. When platelets are stimulated and activated, dense tubules release Ca^2+^ to the cytoplasm, which increases the Ca^2+^ concentration and subsequently activates the cytoskeletal system. Peripheral microtubule coils expand and fold into the cell center, promoting platelet shape change. F-actin polymerization promotes granule release, protein trafficking and activation, and lamellipodia and filopodia extension. EphrinB1-EphA4 EphB1, Sema4D-PlexinB1 CD72 and Sema7A facilitate platelet activation, while PlexinB2 and Slit2-Robo play inhibitory roles in this process.

The regulatory function of NGPs in platelet activation is summarized in **Figure 2**.

## 4 Summary and outlook

In conclusion, platelet formation and activation are complicated and dynamic processes with intensive cytoskeletal system involvement. Actin polymerization provides the mechanical force needed for proplatelet bending and branching. The contraction of platelet actomyosin is critical to limit the proplatelet size. Proplatelet protrusion and elongation rely significantly on microtubules. In the process of platelet activation, actin polymerization is fundamental for filopodia formation and extension and granule secretion, and microtubule expansion enables platelet transformation. As powerful regulators of the intracellular cytoskeletal system, NGPs play fundamental roles in platelet formation and activation, as demonstrated by research reported to date. Sema3A inhibits platelet activation by inhibiting Rac-1 activation and the actin polymerization that typically follows (98). Sema7A promotes platelet granule secretion and integrin activation since it enhances intracellular actin polymerization and cytoskeletal rearrangement (2). Although Sema4D has been demonstrated to facilitate platelet adhesion, aggregation and granule secretion (100), its receptor PlexinB2 exerts an inhibitory effect on platelet activation (101). EphA4 and EphB1 and their common receptor ephrinB1 are expressed on platelets, and ligand–receptor interactions accelerate platelet adhesion, spreading and granule secretion (102, 103). Slit-2 binds to its receptor Robo on platelets and inhibits platelet adhesion, lamellar sheet formation and granule release by downregulating Akt activation (104). Meanwhile, these NGPs have been proven to regulate immune reactions by influencing immune cell adhesion, transmigration and activation.

These established modulatory functions of NGPs toward platelets and inflammation point to their clinical application in hematological and inflammatory diseases. For instance, Sema7A promotes platelet activation in myocardial ischemia and reperfusion and simultaneously enhances leukocyte extravasation, suggesting Sema7A as a potential therapeutic target for the treatment of thrombo-inflammatory reperfusion injury diseases, such as acute coronary ischemic diseases and stroke. Slit-2 has been proven to inhibit platelet adhesion and granule release (104) and to inhibit lymphocyte and neutrophil recruitment in inflammation (162–165). Therefore, enhancing slit-2 function will significantly suppress thrombo-inflammation in ischemia−reperfusion injury and protect the ischemic organs. We believe that additional studies in this research field will be reported and will provide exciting therapeutic candidates for regulating platelet activation and inflammation in related diseases.

## Author contributions

LT and PR contributed to the conception and design of the review. LT and CL collected and summarized the literature, with LT drafting the initial manuscript. PR revised and optimized the manuscript. CL contributed to the manuscript revision by searching literature for rising topics, writing the answers to the reviewers, and drafting the revised manuscript. All authors contributed to the article and approved the submitted version.

## References

[1] Rubenstein DA Yin W. Platelet-activation mechanisms and vascular remodeling. Compr Physiol (2018) 8(3):1117–56. doi: 10.1002/cphy.c170049 29978900

[2] Kohler D Granja T Volz J Koeppen M Langer HF Hansmann G. Red blood cell-derived semaphorin 7A promotes thrombo-inflammation in myocardial ischemia-reperfusion injury through platelet GPIb. Nat Commun (2020) 11(1):1315. doi: 10.1038/s41467-020-14958-x 32161256 PMC7066172

[3] Xu XR Yousef GM Ni H. Cancer and platelet crosstalk: opportunities and challenges for aspirin and other antiplatelet agents. Blood (2018) 131(16):1777–89. doi: 10.1182/blood-2017-05-743187 29519806

[4] Tanguay JF Geoffroy P Sirois MG Libersan D Kumar A Schaub RG. Prevention of in-stent restenosis *via* reduction of thrombo-inflammatory reactions with recombinant p-selectin glycoprotein ligand-1. Thromb Haemost (2004) 91(6):1186–93. doi: 10.1160/TH03-11-0701 15175806

[5] Herr N Mauler M Bode C Duerschmied D. Intravital microscopy of leukocyte-endothelial and platelet-leukocyte interactions in mesenterial veins in mice. J Vis Exp (2015) 13 (102):e53077. doi: 10.3791/53077 PMC4692430 26325284

[6] Totani L Evangelista V. Platelet-leukocyte interactions in cardiovascular disease and beyond. Arterioscler Thromb Vasc Biol (2010) 30(12):2357–61. doi: 10.1161/ATVBAHA.110.207480 PMC3076621 21071701

[7] Franks ZG Campbell RA Weyrich AS Rondina MT. Platelet-leukocyte interactions link inflammatory and thromboembolic events in ischemic stroke. Ann N Y Acad Sci (2010) 1207:11–7. doi: 10.1111/j.1749-6632.2010.05733.x PMC3245960 20955420

[8] Stoll G Nieswandt B. Thrombo-inflammation in acute ischaemic stroke - implications for treatment. Nat Rev Neurol (2019) 15(8):473–81. doi: 10.1038/s41582-019-0221-1 31263257

[9] Kourtzelis I Kotlabova K Lim JH Mitroulis I Ferreira A Chen LS. Developmental endothelial locus-1 modulates platelet-monocyte interactions and instant blood-mediated inflammatory reaction in islet transplantation. Thromb Haemost (2016) 115(4):781–8. doi: 10.1160/TH15-05-0429 PMC4818166 26676803

[10] Seizer P Gawaz M May AE. Platelet-monocyte interactions–a dangerous liaison linking thrombosis, inflammation and atherosclerosis. Curr Med Chem (2008) 15(20):1976–80. doi: 10.2174/092986708785132852 18691053

[11] Dixon DA Tolley ND Bemis-Standoli K Martinez ML Weyrich AS Morrow JD. Expression of COX-2 in platelet-monocyte interactions occurs *via* combinatorial regulation involving adhesion and cytokine signaling. J Clin Invest (2006) 116(10):2727–38. doi: 10.1172/JCI27209. PMC1570372 16998585

[12] Weerasinghe A Athanasiou T Philippidis P Day J Mandal K Warren O. Platelet-monocyte pro-coagulant interactions in on-pump coronary surgery. Eur J Cardiothorac Surg (2006) 29(3):312–8. doi: 10.1016/j.ejcts.2005.11.036 16423536

[13] Aviram M. LDL-platelet interaction under oxidative stress induces macrophage foam cell formation. Thromb Haemost (1995) 74(1):560–4. doi: 10.1055/s-0038-1642738 8578524

[14] Song N Pan K Chen L Jin K. Platelet derived vesicles enhance the TGF-beta signaling pathway of M1 macrophage. Front Endocrinol (Lausanne) (2022) 13:868893. doi: 10.3389/fendo.2022.868893 35370988 PMC8972998

[15] Park G Qian W Zhang MJ Chen YH Ma LW Zeng N. Platelet-rich plasma regulating the repair of ultraviolet b-induced acute tissue inflammation: adjusting macrophage polarization through the activin receptor-follistatin system. Bioengineered (2021) 12(1):3125–36. doi: 10.1080/21655979.2021.1944026 PMC8806634 34193023

[16] Xiong Z Wang Q Li W Huang L Zhang J Zhu J. Platelet-derived growth factor-d activates complement system to propagate macrophage polarization and neovascularization. Front Cell Dev Biol (2021) 9:686886. doi: 10.3389/fcell.2021.686886 34150781 PMC8207142

[17] Uchiyama R Toyoda E Maehara M Wasai S Omura H Watanabe M. Effect of platelet-rich plasma on M1/M2 macrophage polarization. Int J Mol Sci (2021) 22(5):2336. doi: 10.3390/ijms22052336 33652994 PMC7956636

[18] Heffron SP Weinstock A Scolaro B Chen S Sansbury BE Marecki G. Platelet-conditioned media induces an anti-inflammatory macrophage phenotype through EP4. J Thromb Haemost (2021) 19(2):562–73. doi: 10.1111/jth.15172 PMC7902474 33171016

[19] Laffont B Corduan A Rousseau M Duchez AC Lee CH Boilard E. Platelet microparticles reprogram macrophage gene expression and function. Thromb Haemost (2016) 115(2):311–23. doi: 10.1160/TH15-05-0389 26333874

[20] Engelmann B Massberg S. Thrombosis as an intravascular effector of innate immunity. Nat Rev Immunol (2013) 13(1):34–45. doi: 10.1038/nri3345 23222502

[21] Martinod K Deppermann C. Immunothrombosis and thromboinflammation in host defense and disease. Platelets (2021) 32(3):314–24. doi: 10.1080/09537104.2020.1817360 32896192

[22] Mandel J Casari M Stepanyan M Martyanov A Deppermann C. Beyond hemostasis: platelet innate immune interactions and thromboinflammation. Int J Mol Sci (2022) 23(7):3868. doi: 10.3390/ijms23073868 35409226 PMC8998935

[23] Kolodkin AL Matthes DJ Goodman CS. The semaphorin genes encode a family of transmembrane and secreted growth cone guidance molecules. Cell (1993) 75(7):1389–99. doi: 10.1016/0092-8674(93)90625-Z 8269517

[24] Limoni G. Modelling and refining neuronal circuits with guidance cues: involvement of semaphorins. Int J Mol Sci (2021) 22(11):6111. doi: 10.3390/ijms22116111 34204060 PMC8201269

[25] Xie Z Huganir RL Penzes P. Activity-dependent dendritic spine structural plasticity is regulated by small GTPase Rap1 and its target AF-6. Neuron (2005) 48(4):605–18. doi: 10.1016/j.neuron.2005.09.027 16301177

[26] Vanderhaeghen P Cheng HJ. Guidance molecules in axon pruning and cell death. Cold Spring Harb Perspect Biol (2010) 2(6):a001859. doi: 10.1101/cshperspect.a001859 20516131 PMC2869516

[27] Chabbert-de Ponnat I Marie-Cardine A Pasterkamp RJ Schiavon V Tamagnone L Thomasset N. Soluble CD100 functions on human monocytes and immature dendritic cells require plexin C1 and plexin B1, respectively. Int Immunol (2005) 17(4):439–47. doi: 10.1093/intimm/dxh224 15746246

[28] Ishida I Kumanogoh A Suzuki K Akahani S Noda K Kikutani H. Involvement of CD100, a lymphocyte semaphorin, in the activation of the human immune system *via* CD72: implications for the regulation of immune and inflammatory responses. Int Immunol (2003) 15(8):1027–34. doi: 10.1093/intimm/dxg098 12882840

[29] Mirakaj V Rosenberger P. Immunomodulatory functions of neuronal guidance proteins. Trends Immunol (2017) 38(6):444–56. doi: 10.1016/j.it.2017.03.007 28438491

[30] Basile JR Barac A Zhu T Guan KL Gutkind JS. Class IV semaphorins promote angiogenesis by stimulating rho-initiated pathways through plexin-b. Cancer Res (2004) 64(15):5212–24. doi: 10.1158/0008-5472.CAN-04-0126 15289326

[31] Sun Q Zhou H Binmadi NO Basile JR. Hypoxia-inducible factor-1-mediated regulation of semaphorin 4D affects tumor growth and vascularity. J Biol Chem (2009) 284(46):32066–74. doi: 10.1074/jbc.M109.057166 PMC2797277 19762474

[32] Choi ES Nichol JL Hokom MM Hornkohl AC Hunt P. Platelets generated *in vitro* from proplatelet-displaying human megakaryocytes are functional. Blood (1995) 85(2):402–13. doi: 10.1182/blood.V85.2.402.402 7529062

[33] Cramer EM Norol F Guichard J Breton-Gorius J Vainchenker W Masse JM. Ultrastructure of platelet formation by human megakaryocytes cultured with the mpl ligand. Blood (1997) 89(7):2336–46. doi: 10.1182/blood.V89.7.2336 9116277

[34] Zimmet J Ravid K. Polyploidy: occurrence in nature, mechanisms, and significance for the megakaryocyte-platelet system. Exp Hematol (2000) 28(1):3–16. doi: 10.1016/S0301-472X(99)00124-1 10658672

[35] Gurney AL Carver-Moore K de Sauvage FJ Moore MW. Thrombocytopenia in c-mpl-deficient mice. Science (1994) 265(5177):1445–7. doi: 10.1126/science.8073287 8073287

[36] Eckly A Heijnen H Pertuy F Geerts W Proamer F Rinckel JY. Biogenesis of the demarcation membrane system (DMS) in megakaryocytes. Blood (2014) 123(6):921–30. doi: 10.1182/blood-2013-03-492330 24152908

[37] Italiano JE Jr. Lecine P Shivdasani RA Hartwig JH. Blood platelets are assembled principally at the ends of proplatelet processes produced by differentiated megakaryocytes. J Cell Biol (1999) 147(6):1299–312. doi: 10.1083/jcb.147.6.1299 PMC2168104 10601342

[38] Junt T Schulze H Chen Z Massberg S Goerge T Krueger A. Dynamic visualization of thrombopoiesis within bone marrow. Science (2007) 317(5845):1767–70. doi: 10.1126/science.1146304 17885137

[39] Patel SR Richardson JL Schulze H Kahle E Galjart N Drabek K. Differential roles of microtubule assembly and sliding in proplatelet formation by megakaryocytes. Blood (2005) 106(13):4076–85. doi: 10.1182/blood-2005-06-2204 PMC1895246 16118321

[40] Handagama PJ Feldman BF Jain NC Farver TB Kono CS. Circulating proplatelets: isolation and quantitation in healthy rats and in rats with induced acute blood loss. Am J Vet Res (1987) 48(6):962–5. 3605813

[41] Howell WH Donahue DD. The production of blood platelets in the lungs. J Exp Med (1937) 65(2):177–203. doi: 10.1084/jem.65.2.177 19870594 PMC2133484

[42] Theodoropoulos PA Gravanis A Tsapara A Margioris AN Papadogiorgaki E Galanopoulos V. Cytochalasin b may shorten actin filaments by a mechanism independent of barbed end capping. Biochem Pharmacol (1994) 47(10):1875–81. doi: 10.1016/0006-2952(94)90318-2 8204105

[43] Spudich JA Lin S. Cytochalasin b, its interaction with actin and actomyosin from muscle (cell movement-microfilaments-rabbit striated muscle). Proc Natl Acad Sci USA (1972) 69(2):442–6. doi: 10.1073/pnas.69.2.442 PMC426476 4258316

[44] Weiss A Schiaffino S Leinwand LA. Comparative sequence analysis of the complete human sarcomeric myosin heavy chain family: implications for functional diversity. J Mol Biol (1999) 290(1):61–75. doi: 10.1006/jmbi.1999.2865 10388558

[45] Kunishima S Kojima T Matsushita T Tanaka T Tsurusawa M Furukawa Y. Mutations in the NMMHC-a gene cause autosomal dominant macrothrombocytopenia with leukocyte inclusions (May-hegglin anomaly/Sebastian syndrome). Blood (2001) 97(4):1147–9. doi: 10.1182/blood.V97.4.1147 11159552

[46] Seri M Cusano R Gangarossa S Caridi G Bordo D Lo Nigro C. Mutations in MYH9 result in the may-hegglin anomaly, and fechtner and Sebastian syndromes. May-Heggllin/Fechtner Syndrome Consortium Nat Genet (2000) 26(1):103–5. doi: 10.1038/79063 10973259

[47] Kelley MJ Jawien W Ortel TL Korczak JF. Mutation of MYH9, encoding non-muscle myosin heavy chain a, in may-hegglin anomaly. Nat Genet (2000) 26(1):106–8. doi: 10.1038/79069 10973260

[48] Heath KE Campos-Barros A Toren A Rozenfeld-Granot G Carlsson LE Savige J. Nonmuscle myosin heavy chain IIA mutations define a spectrum of autosomal dominant macrothrombocytopenias: may-hegglin anomaly and fechtner, Sebastian, Epstein, and alport-like syndromes. Am J Hum Genet (2001) 69(5):1033–45. doi: 10.1086/324267 PMC1274350 11590545

[49] Althaus K Najm J Greinacher A. MYH9 related platelet disorders - often unknown and misdiagnosed. Klin Padiatr (2011) 223(3):120–5. doi: 10.1055/s-0031-1275664 21567368

[50] Seri M Pecci A Di Bari F Cusano R Savino M Panza E. MYH9-related disease: may-hegglin anomaly, Sebastian syndrome, fechtner syndrome, and Epstein syndrome are not distinct entities but represent a variable expression of a single illness. Med (Baltimore) (2003) 82(3):203–15. doi: 10.1097/01.md.0000076006.64510.5c 12792306

[51] Vicente-Manzanares M Ma X Adelstein RS Horwitz AR. Non-muscle myosin II takes centre stage in cell adhesion and migration. Nat Rev Mol Cell Biol (2009) 10(11):778–90. doi: 10.1038/nrm2786 PMC2834236 19851336

[52] Aslan JE McCarty OJ. Rho GTPases in platelet function. J Thromb Haemost (2013) 11(1):35–46. doi: 10.1111/jth.12051 23121917 PMC3928789

[53] Pleines I Cherpokova D Bender M. Rho GTPases and their downstream effectors in megakaryocyte biology. Platelets (2019) 30(1):9–16. doi: 10.1080/09537104.2018.1478071 29913074

[54] Pleines I Hagedorn I Gupta S May F Chakarova L van Hengel J. Megakaryocyte-specific RhoA deficiency causes macrothrombocytopenia and defective platelet activation in hemostasis and thrombosis. Blood (2012) 119(4):1054–63. doi: 10.1182/blood-2011-08-372193 22045984

[55] Jaffe AB Hall A. Rho GTPases: biochemistry and biology. Annu Rev Cell Dev Biol (2005) 21:247–69. doi: 10.1146/annurev.cellbio.21.020604.150721 16212495

[56] Chang Y Aurade F Larbret F Zhang Y Le Couedic JP Momeux L. Proplatelet formation is regulated by the Rho/ROCK pathway. Blood (2007) 109(10):4229–36. doi: 10.1182/blood-2006-04-020024 17244674

[57] Chen Z Naveiras O Balduini A Mammoto A Conti MA Adelstein RS. The may-hegglin anomaly gene MYH9 is a negative regulator of platelet biogenesis modulated by the rho-ROCK pathway. Blood (2007) 110(1):171–9. doi: 10.1182/blood-2007-02-071589 PMC1896110 17392504

[58] Dutting S Gaits-Iacovoni F Stegner D Popp M Antkowiak A van Eeuwijk JMM. A Cdc42/RhoA regulatory circuit downstream of glycoprotein ib guides transendothelial platelet biogenesis. Nat Commun (2017) 8:15838. doi: 10.1038/ncomms15838 28643773 PMC5481742

[59] Handagama PJ Feldman BF Jain NC Farver TB Kono CS. *In vitro* platelet release by rat megakaryocytes: effect of metabolic inhibitors and cytoskeletal disrupting agents. Am J Vet Res (1987) 48(7):1142–6. 3631701

[60] Kunishima S Kobayashi R Itoh TJ Hamaguchi M Saito H. Mutation of the beta1-tubulin gene associated with congenital macrothrombocytopenia affecting microtubule assembly. Blood (2009) 113(2):458–61. doi: 10.1182/blood-2008-06-162610 18849486

[61] Lecine P Italiano JE Jr. Kim SW Villeval JL Shivdasani RA. Hematopoietic-specific beta 1 tubulin participates in a pathway of platelet biogenesis dependent on the transcription factor NF-E2. Blood (2000) 96(4):1366–73. doi: 10.1182/blood.V96.4.1366 10942379

[62] Fernandez DI Kuijpers MJE Heemskerk JWM. Platelet calcium signaling by G-protein coupled and ITAM-linked receptors regulating anoctamin-6 and procoagulant activity. Platelets (2021) 32(7):863–71. doi: 10.1080/09537104.2020.1859103 33356720

[63] Offermanns S. Activation of platelet function through G protein-coupled receptors. Circ Res (2006) 99(12):1293–304. doi: 10.1161/01.RES.0000251742.71301.16 17158345

[64] Lhermusier T Chap H Payrastre B. Platelet membrane phospholipid asymmetry: from the characterization of a scramblase activity to the identification of an essential protein mutated in Scott syndrome. J Thromb Haemost (2011) 9(10):1883–91. doi: 10.1111/j.1538-7836.2011.04478.x 21958383

[65] O'Donnell VB Murphy RC Watson SP. Platelet lipidomics: modern day perspective on lipid discovery and characterization in platelets. Circ Res (2014) 114(7):1185–203. doi: 10.1161/CIRCRESAHA.114.301597 PMC4021279 24677238

[66] Thon JN Italiano JE. Platelets: production, morphology and ultrastructure. Handb Exp Pharmacol (2012) 210):3–22. doi: 10.1007/978-3-642-29423-5_1 22918725

[67] Abou-Saleh H Theoret JF Yacoub D Merhi Y. Neutrophil p-selectin-glycoprotein-ligand-1 binding to platelet p-selectin enhances metalloproteinase 2 secretion and platelet-neutrophil aggregation. Thromb Haemost (2005) 94(6):1230–5. doi: 10.1160/TH05-05-0344 16411399

[68] Lam FW Da Q Guillory B Cruz MA. Recombinant human vimentin binds to p-selectin and blocks neutrophil capture and rolling on platelets and endothelium. J Immunol (2018) 200(5):1718–26. doi: 10.4049/jimmunol.1700784 PMC5821592 29335256

[69] Folkman J Browder T Palmblad J. Angiogenesis research: guidelines for translation to clinical application. Thromb Haemost (2001) 86(1):23–33. 11487011

[70] Weibrich G Kleis WK Hafner G. Growth factor levels in the platelet-rich plasma produced by 2 different methods: curasan-type PRP kit versus PCCS PRP system. Int J Oral Maxillofac Implants (2002) 17(2):184–90. 11958400

[71] Sundman EA Cole BJ Fortier LA. Growth factor and catabolic cytokine concentrations are influenced by the cellular composition of platelet-rich plasma. Am J Sports Med (2011) 39(10):2135–40. doi: 10.1177/0363546511417792 21846925

[72] Denis CV Andre P Saffaripour S Wagner DD. Defect in regulated secretion of p-selectin affects leukocyte recruitment in von willebrand factor-deficient mice. Proc Natl Acad Sci USA (2001) 98(7):4072–7. doi: 10.1073/pnas.061307098 PMC31181 11274431

[73] Du X. Signaling and regulation of the platelet glycoprotein ib-IX-V complex. Curr Opin Hematol (2007) 14(3):262–9. doi: 10.1097/MOH.0b013e3280dce51a 17414217

[74] Woods VL Jr. Wolff LE Keller DM. Resting platelets contain a substantial centrally located pool of glycoprotein IIb-IIIa complex which may be accessible to some but not other extracellular proteins. J Biol Chem (1986) 261(32):15242–51. doi: 10.1016/S0021-9258(18)66859-0 2429967

[75] Gremmel T Frelinger AL 3rd Michelson AD. Platelet physiology. Semin Thromb Hemost (2016) 42(3):191–204. doi: 10.1055/s-0035-1564835 26926581

[76] Woronowicz K Dilks JR Rozenvayn N Dowal L Blair PS Peters CG. The platelet actin cytoskeleton associates with SNAREs and participates in alpha-granule secretion. Biochemistry (2010) 49(21):4533–42. doi: 10.1021/bi100541t PMC2892908 20429610

[77] Kiuru J Viinikka L Myllyla G Pesonen K Perheentupa J. Cytoskeleton-dependent release of human platelet epidermal growth factor. Life Sci (1991) 49(26):1997–2003. doi: 10.1016/0024-3205(91)90642-O 1749310

[78] Patel-Hett S Richardson JL Schulze H Drabek K Isaac NA Hoffmeister K. Visualization of microtubule growth in living platelets reveals a dynamic marginal band with multiple microtubules. Blood (2008) 111(9):4605–16. doi: 10.1182/blood-2007-10-118844 PMC2343595 18230754

[79] Schoenwaelder SM Hughan SC Boniface K Fernando S Holdsworth M Thompson PE. RhoA sustains integrin alpha IIbbeta 3 adhesion contacts under high shear. J Biol Chem (2002) 277(17):14738–46. doi: 10.1074/jbc.M200661200 11830597

[80] Akbar H Kim J Funk K Cancelas JA Shang X Chen L. Genetic and pharmacologic evidence that Rac1 GTPase is involved in regulation of platelet secretion and aggregation. J Thromb Haemost (2007) 5(8):1747–55. doi: 10.1111/j.1538-7836.2007.02646.x 17663742

[81] McCarty OJ Larson MK Auger JM Kalia N Atkinson BT Pearce AC. Rac1 is essential for platelet lamellipodia formation and aggregate stability under flow. J Biol Chem (2005) 280(47):39474–84. doi: 10.1074/jbc.M504672200 PMC1395485 16195235

[82] Pula G Poole AW. Critical roles for the actin cytoskeleton and cdc42 in regulating platelet integrin alpha2beta1. Platelets (2008) 19(3):199–210. doi: 10.1080/09537100701777303 18432521

[83] Brass LF. Thrombin and platelet activation. Chest (2003) 124(3 Suppl):18S–25S. doi: 10.1378/chest.124.3_suppl.18S 12970120

[84] Tomaiuolo M Brass LF Stalker TJ. Regulation of platelet activation and coagulation and its role in vascular injury and arterial thrombosis. Interv Cardiol Clin (2017) 6(1):1–12. doi: 10.1016/j.iccl.2016.08.001 27886814 PMC5154246

[85] Jones CI Barrett NE Moraes LA Gibbins JM Jackson DE. Endogenous inhibitory mechanisms and the regulation of platelet function. Methods Mol Biol (2012) 788:341–66. doi: 10.1007/978-1-61779-307-3_23 22130718

[86] Stefanini L Bergmeier W. Negative regulators of platelet activation and adhesion. J Thromb Haemost (2018) 16(2):220–30. doi: 10.1111/jth.13910 PMC5809258 29193689

[87] Pasterkamp RJ Kolodkin AL. SnapShot: axon guidance. Cell (2013) 153(2):494, e1–2. doi: 10.1016/j.cell.2013.03.031 23582334

[88] Kolodkin AL Pasterkamp RJ. SnapShot: axon guidance II. Cell (2013) 153(3):722 e1. doi: 10.1016/j.cell.2013.04.004 23622251

[89] Kolodkin AL Tessier-Lavigne M. Mechanisms and molecules of neuronal wiring: a primer. Cold Spring Harb Perspect Biol (2011) 3(6):a001727. doi: 10.1101/cshperspect.a001727 21123392 PMC3098670

[90] Bashaw GJ Klein R. Signaling from axon guidance receptors. Cold Spring Harb Perspect Biol (2010) 2(5):a001941. doi: 10.1101/cshperspect.a001941 20452961 PMC2857166

[91] Tran TS Kolodkin AL Bharadwaj R. Semaphorin regulation of cellular morphology. Annu Rev Cell Dev Biol (2007) 23:263–92. doi: 10.1146/annurev.cellbio.22.010605.093554 17539753

[92] Lefebvre JL Kostadinov D Chen WV Maniatis T Sanes JR. Protocadherins mediate dendritic self-avoidance in the mammalian nervous system. Nature (2012) 488(7412):517–21. doi: 10.1038/nature11305 PMC3427422 22842903

[93] Stoeckli ET. Understanding axon guidance: are we nearly there yet? Development (2018) 145(10):dev151415. doi: 10.1242/dev.151415 29759980

[94] Nojima S. Class IV semaphorins in disease pathogenesis. Pathol Int (2022) 72(10):471–87. doi: 10.1111/pin.13270 36066011

[95] Wang J Huang Y Zhang J Xing B Xuan W Wang H. NRP-2 in tumor lymphangiogenesis and lymphatic metastasis. Cancer Lett (2018) 418:176–84. doi: 10.1016/j.canlet.2018.01.040 29339213

[96] Verlinden L Vanderschueren D Verstuyf A. Semaphorin signaling in bone. Mol Cell Endocrinol (2016) 432:66–74. doi: 10.1016/j.mce.2015.09.009 26365296

[97] Suchting S Bicknell R Eichmann A. Neuronal clues to vascular guidance. Exp Cell Res (2006) 312(5):668–75. doi: 10.1016/j.yexcr.2005.11.009 16330027

[98] Kashiwagi H Shiraga M Kato H Kamae T Yamamoto N Tadokoro S. Negative regulation of platelet function by a secreted cell repulsive protein, semaphorin 3A. Blood (2005) 106(3):913–21. doi: 10.1182/blood-2004-10-4092 15831706

[99] Jaimes Y Gras C Goudeva L Buchholz S Eiz-Vesper B Seltsam A. Semaphorin 7A inhibits platelet production from CD34+ progenitor cells. J Thromb Haemost (2012) 10(6):1100–8. doi: 10.1111/j.1538-7836.2012.04708.x 22448926

[100] Zhu L Bergmeier W Wu J Jiang H Stalker TJ Cieslak M. Regulated surface expression and shedding support a dual role for semaphorin 4D in platelet responses to vascular injury. Proc Natl Acad Sci USA (2007) 104(5):1621–6. doi: 10.1073/pnas.0606344104 PMC1785259 17244710

[101] Garcia A Dunoyer-Geindre S Zapilko V Nolli S Reny JL Fontana P. Functional validation of microRNA-126-3p as a platelet reactivity regulator using human haematopoietic stem cells. Thromb Haemost (2019) 119(2):254–63. doi: 10.1055/s-0038-1676802 30602197

[102] Prevost N Woulfe D Tanaka T Brass LF. Interactions between eph kinases and ephrins provide a mechanism to support platelet aggregation once cell-to-cell contact has occurred. Proc Natl Acad Sci USA (2002) 99(14):9219–24. doi: 10.1073/pnas.142053899 PMC123121 12084815

[103] Prevost N Woulfe DS Tognolini M Tanaka T Jian W Fortna RR. Signaling by ephrinB1 and eph kinases in platelets promotes Rap1 activation, platelet adhesion, and aggregation *via* effector pathways that do not require phosphorylation of ephrinB1. Blood (2004) 103(4):1348–55. doi: 10.1182/blood-2003-06-1781 14576067

[104] Patel S Huang YW Reheman A Pluthero FG Chaturvedi S Mukovozov IM. The cell motility modulator Slit2 is a potent inhibitor of platelet function. Circulation (2012) 126(11):1385–95. doi: 10.1161/CIRCULATIONAHA.112.105452 22865890

[105] Nishide M Nojima S Ito D Takamatsu H Koyama S Kang S. Semaphorin 4D inhibits neutrophil activation and is involved in the pathogenesis of neutrophil-mediated autoimmune vasculitis. Ann Rheum Dis (2017) 76(8):1440–8. doi: 10.1136/annrheumdis-2016-210706 PMC5738596 28416516

[106] Kolodkin AL Levengood DV Rowe EG Tai YT Giger RJ Ginty DD. Neuropilin is a semaphorin III receptor. Cell (1997) 90(4):753–62. doi: 10.1016/S0092-8674(00)80535-8 9288754

[107] Tamagnone L Comoglio PM. Signalling by semaphorin receptors: cell guidance and beyond. Trends Cell Biol (2000) 10(9):377–83. doi: 10.1016/S0962-8924(00)01816-X 10932095

[108] Kiseleva EP Rutto KV. Semaphorin 3A in the immune system: twenty years of study. Biochem (Mosc) (2022) 87(7):640–57. doi: 10.1134/S0006297922070069 PMC9282903 36154881

[109] Kawasaki T Bekku Y Suto F Kitsukawa T Taniguchi M Nagatsu I. Requirement of neuropilin 1-mediated Sema3A signals in patterning of the sympathetic nervous system. Development (2002) 129(3):671–80. doi: 10.1242/dev.129.3.671 11830568

[110] National Center for Biotechnology Information (NCBI). National center for biotechnology information. Bethesda (MD: National Library of Medicine (US (1988). Available at: https://www.ncbi.nlm.nih.gov/.

[111] Catalano A Caprari P Moretti S Faronato M Tamagnone L Procopio A. Semaphorin-3A is expressed by tumor cells and alters T-cell signal transduction and function. Blood (2006) 107(8):3321–9. doi: 10.1182/blood-2005-06-2445 16380453

[112] Lepelletier Y Moura IC Hadj-Slimane R Renand A Fiorentino S Baude C. Immunosuppressive role of semaphorin-3A on T cell proliferation is mediated by inhibition of actin cytoskeleton reorganization. Eur J Immunol (2006) 36(7):1782–93. doi: 10.1002/eji.200535601 16791896

[113] Cozacov R Halasz K Haj T Vadasz Z. Semaphorin 3A: is a key player in the pathogenesis of asthma. Clin Immunol (2017) 184:70–2. doi: 10.1016/j.clim.2017.05.011 28502680

[114] Guo Q Ma XX Gao H Shi LJ Zhong YC Xie LF. Association of semaphorin 3A with thrombocytopenia in systemic lupus erythematosus. Beijing Da Xue Xue Bao Yi Xue Ban (2020) 52(5):892–6. doi: 10.19723/j.issn.1671-167X.2020.05.016 PMC7653423 33047725

[115] Liu H Juo ZS Shim AH Focia PJ Chen X Garcia KC. Structural basis of semaphorin-plexin recognition and viral mimicry from Sema7A and A39R complexes with PlexinC1. Cell (2010) 142(5):749–61. doi: 10.1016/j.cell.2010.07.040 PMC2936782 20727575

[116] Pasterkamp RJ Peschon JJ Spriggs MK Kolodkin AL. Semaphorin 7A promotes axon outgrowth through integrins and MAPKs. Nature (2003) 424(6947):398–405. doi: 10.1038/nature01790 12879062

[117] Tamagnone L Artigiani S Chen H He Z Ming GI Song H. Plexins are a large family of receptors for transmembrane, secreted, and GPI-anchored semaphorins in vertebrates. Cell (1999) 99(1):71–80. doi: 10.1016/S0092-8674(00)80063-X 10520995

[118] Morote-Garcia JC Napiwotzky D Kohler D Rosenberger P. Endothelial semaphorin 7A promotes neutrophil migration during hypoxia. Proc Natl Acad Sci USA (2012) 109(35):14146–51. doi: 10.1073/pnas.1202165109 PMC3435204 22891341

[119] Suzuki K Okuno T Yamamoto M Pasterkamp RJ Takegahara N Takamatsu H. Semaphorin 7A initiates T-cell-mediated inflammatory responses through alpha1beta1 integrin. Nature (2007) 446(7136):680–4. doi: 10.1038/nature05652 17377534

[120] Zhang M Wang L Dong M Li Z Jin F. Endothelial semaphorin 7A promotes inflammation in seawater aspiration-induced acute lung injury. Int J Mol Sci (2014) 15(11):19650–61. doi: 10.3390/ijms151119650 PMC4264131 25353180

[121] Massberg S Gawaz M Gruner S Schulte V Konrad I Zohlnhofer D. A crucial role of glycoprotein VI for platelet recruitment to the injured arterial wall in vivo. J Exp Med (2003) 197(1):41–9. doi: 10.1084/jem.20020945 PMC2193801 12515812

[122] Civin CI Strauss LC Brovall C Fackler MJ Schwartz JF Shaper JH. Antigenic analysis of hematopoiesis. III. a hematopoietic progenitor cell surface antigen defined by a monoclonal antibody raised against KG-1a cells. J Immunol (1984) 133(1):157–65. 6586833

[123] Safi W Kuehnl A Nussler A Eckstein HH Pelisek J. Differentiation of human CD14+ monocytes: an experimental investigation of the optimal culture medium and evidence of a lack of differentiation along the endothelial line. Exp Mol Med (2016) 48(4):e227. doi: 10.1038/emm.2016.11 27080367 PMC4855273

[124] Moretti S Procopio A Boemi M Catalano A. Neuronal semaphorins regulate a primary immune response. Curr Neurovasc Res (2006) 3(4):295–305. doi: 10.2174/156720206778792939 17109625

[125] Suzuki K Kumanogoh A Kikutani H. CD100/Sema4D, a lymphocyte semaphorin involved in the regulation of humoral and cellular immune responses. Cytokine Growth Factor Rev (2003) 14(1):17–24. doi: 10.1016/S1359-6101(02)00073-4 12485616

[126] Maleki KT Cornillet M Bjorkstrom NK. Soluble SEMA4D/CD100: a novel immunoregulator in infectious and inflammatory diseases. Clin Immunol (2016) 163:52–9. doi: 10.1016/j.clim.2015.12.012 26732857

[127] Hayashi M Nakashima T. Semaphorin and osteoporosis. Clin Calcium (2016) 26(10):1419–27. 27666689

[128] Bougeret C Mansur IG Dastot H Schmid M Mahouy G Bensussan A. Increased surface expression of a newly identified 150-kDa dimer early after human T lymphocyte activation. J Immunol (1992) 148(2):318–23. doi: 10.4049/jimmunol.148.2.318 1530858

[129] Love CA Harlos K Mavaddat N Davis SJ Stuart DI Jones EY. The ligand-binding face of the semaphorins revealed by the high-resolution crystal structure of SEMA4D. Nat Struct Biol (2003) 10(10):843–8. doi: 10.1038/nsb977 12958590

[130] Mou P Zeng Z Li Q Liu X Xin X Wannemacher KM. Identification of a calmodulin-binding domain in Sema4D that regulates its exodomain shedding in platelets. Blood (2013) 121(20):4221–30. doi: 10.1182/blood-2012-11-470609 PMC3656454 23564909

[131] Kumanogoh A Kikutani H. Immunological functions of the neuropilins and plexins as receptors for semaphorins. Nat Rev Immunol (2013) 13(11):802–14. doi: 10.1038/nri3545 24319778

[132] Li M O'Sullivan KM Jones LK Lo C Semple T Kumanogoh A. Endogenous CD100 promotes glomerular injury and macrophage recruitment in experimental crescentic glomerulonephritis. Immunology (2009) 128(1):114–22. doi: 10.1111/j.1365-2567.2009.03098.x PMC2747144 19689741

[133] Wannemacher KM Zhu L Jiang H Fong KP Stalker TJ Lee D. Diminished contact-dependent reinforcement of syk activation underlies impaired thrombus growth in mice lacking semaphorin 4D. Blood (2010) 116(25):5707–15. doi: 10.1182/blood-2010-04-279943 PMC3031415 20855865

[134] Zhu L Stalker TJ Fong KP Jiang H Tran A Crichton I. Disruption of SEMA4D ameliorates platelet hypersensitivity in dyslipidemia and confers protection against the development of atherosclerosis. Arterioscler Thromb Vasc Biol (2009) 29(7):1039–45. doi: 10.1161/ATVBAHA.109.185405 PMC2877695 19390055

[135] Faruki S Geahlen RL Asai DJ. Syk-dependent phosphorylation of microtubules in activated b-lymphocytes. J Cell Sci (2000) 113(Pt 14):2557–65. doi: 10.1242/jcs.113.14.2557 10862713

[136] Sada K Minami Y Yamamura H. Relocation of syk protein-tyrosine kinase to the actin filament network and subsequent association with fak. Eur J Biochem (1997) 248(3):827–33. doi: 10.1111/j.1432-1033.1997.00827.x 9342235

[137] Peters JD Furlong MT Asai DJ Harrison ML Geahlen RL. Syk, activated by cross-linking the b-cell antigen receptor, localizes to the cytosol where it interacts with and phosphorylates alpha-tubulin on tyrosine. J Biol Chem (1996) 271(9):4755–62. doi: 10.1074/jbc.271.9.4755 8617742

[138] Akkerman JW. From low-density lipoprotein to platelet activation. Int J Biochem Cell Biol (2008) 40(11):2374–8. doi: 10.1016/j.biocel.2008.04.002 18468940

[139] Andrews RK Munday AD Mitchell CA Berndt MC. Interaction of calmodulin with the cytoplasmic domain of the platelet membrane glycoprotein ib-IX-V complex. Blood (2001) 98(3):681–7. doi: 10.1182/blood.V98.3.681 11468167

[140] Andrews RK Suzuki-Inoue K Shen Y Tulasne D Watson SP Berndt MC. Interaction of calmodulin with the cytoplasmic domain of platelet glycoprotein VI. Blood (2002) 99(11):4219–21. doi: 10.1182/blood-2001-11-0008 12010829

[141] Wong MX Harbour SN Wee JL Lau LM Andrews RK Jackson DE. Proteolytic cleavage of platelet endothelial cell adhesion molecule-1 (PECAM-1/CD31) is regulated by a calmodulin-binding motif. FEBS Lett (2004) 568(1-3):70–8. doi: 10.1016/j.febslet.2004.04.094 15196923

[142] Ple H Landry P Benham A Coarfa C Gunaratne PH Provost P. The repertoire and features of human platelet microRNAs. PloS One (2012) 7(12):e50746. doi: 10.1371/journal.pone.0050746 23226537 PMC3514217

[143] Sunderland N Skroblin P Barwari T Huntley RP Lu R Joshi A. MicroRNA biomarkers and platelet reactivity: the clot thickens. Circ Res (2017) 120(2):418–35. doi: 10.1161/CIRCRESAHA.116.309303 28104774

[144] Landry P Plante I Ouellet DL Perron MP Rousseau G Provost P. Existence of a microRNA pathway in anucleate platelets. Nat Struct Mol Biol (2009) 16(9):961–6. doi: 10.1038/nsmb.1651 PMC2911476 19668211

[145] Ts'ao CH. Rough endoplasmic reticulum and ribosomes in blood platelets. Scand J Haematol (1971) 8(2):134–40. doi: 10.1111/j.1600-0609.1971.tb01964.x 5094954

[146] Bray PF McKenzie SE Edelstein LC Nagalla S Delgrosso K Ertel A. The complex transcriptional landscape of the anucleate human platelet. BMC Genomics (2013) 14:1. doi: 10.1186/1471-2164-14-1 23323973 PMC3722126

[147] Rowley JW Oler AJ Tolley ND Hunter BN Low EN Nix DA. Genome-wide RNA-seq analysis of human and mouse platelet transcriptomes. Blood (2011) 118(14):e101–11. doi: 10.1182/blood-2011-03-339705 PMC3193274 21596849

[148] Kieffer N Guichard J Farcet JP Vainchenker W Breton-Gorius J. Biosynthesis of major platelet proteins in human blood platelets. Eur J Biochem (1987) 164(1):189–95. doi: 10.1111/j.1432-1033.1987.tb11010.x 3830180

[149] Wilkinson DG. Eph receptors and ephrins: regulators of guidance and assembly. Int Rev Cytol (2000) 196:177–244. doi: 10.1016/S0074-7696(00)96005-4 10730216

[150] Coulthard MG Morgan M Woodruff TM Arumugam TV Taylor SM Carpenter TC. Eph/Ephrin signaling in injury and inflammation. Am J Pathol (2012) 181(5):1493–503. doi: 10.1016/j.ajpath.2012.06.043 23021982

[151] Chan B Sukhatme VP. Receptor tyrosine kinase EphA2 mediates thrombin-induced upregulation of ICAM-1 in endothelial cells in vitro. Thromb Res (2009) 123(5):745–52. doi: 10.1016/j.thromres.2008.07.010 PMC2684450 18768213

[152] Fang WB Ireton RC Zhuang G Takahashi T Reynolds A Chen J. Overexpression of EPHA2 receptor destabilizes adherens junctions via a RhoA-dependent mechanism. J Cell Sci (2008) 121(Pt 3):358–68. doi: 10.1242/jcs.017145 18198190

[153] Pitulescu ME Adams RH. Eph/ephrin molecules–a hub for signaling and endocytosis. Genes Dev (2010) 24(22):2480–92. doi: 10.1101/gad.1973910 PMC2975924 21078817

[154] Liu X Hawkes E Ishimaru T Tran T Sretavan DW. EphB3: an endogenous mediator of adult axonal plasticity and regrowth after CNS injury. J Neurosci (2006) 26(12):3087–101. doi: 10.1523/JNEUROSCI.4797-05.2006 PMC6674090 16554460

[155] Prevost N Woulfe DS Jiang H Stalker TJ Marchese P Ruggeri ZM. Eph kinases and ephrins support thrombus growth and stability by regulating integrin outside-in signaling in platelets. Proc Natl Acad Sci USA (2005) 102(28):9820–5. doi: 10.1073/pnas.0404065102 PMC1174973 15994237

[156] Kidd T Brose K Mitchell KJ Fetter RD Tessier-Lavigne M Goodman CS. Roundabout controls axon crossing of the CNS midline and defines a novel subfamily of evolutionarily conserved guidance receptors. Cell (1998) 92(2):205–15. doi: 10.1016/S0092-8674(00)80915-0 9458045

[157] Kidd T Bland KS Goodman CS. Slit is the midline repellent for the robo receptor in drosophila. Cell (1999) 96(6):785–94. doi: 10.1016/S0092-8674(00)80589-9 10102267

[158] Chaturvedi S Robinson LA. Slit2-robo signaling in inflammation and kidney injury. Pediatr Nephrol (2015) 30(4):561–6. doi: 10.1007/s00467-014-2825-4 24777535

[159] Qian L Liu J Bodmer R. Slit and robo control cardiac cell polarity and morphogenesis. Curr Biol (2005) 15(24):2271–8. doi: 10.1016/j.cub.2005.10.037 16360689

[160] Xian J Clark KJ Fordham R Pannell R Rabbitts TH Rabbitts PH. Inadequate lung development and bronchial hyperplasia in mice with a targeted deletion in the Dutt1/Robo1 gene. Proc Natl Acad Sci USA (2001) 98(26):15062–6. doi: 10.1073/pnas.251407098 PMC64983 11734623

[161] Grieshammer U Le M Plump AS Wang F Tessier-Lavigne M Martin GR. SLIT2-mediated ROBO2 signaling restricts kidney induction to a single site. Dev Cell (2004) 6(5):709–17. doi: 10.1016/S1534-5807(04)00108-X 15130495

[162] Wu JY Feng L Park HT Havlioglu N Wen L Tang H. The neuronal repellent slit inhibits leukocyte chemotaxis induced by chemotactic factors. Nature (2001) 410(6831):948–52. doi: 10.1038/35073616 PMC2072862 11309622

[163] Tole S Mukovozov IM Huang YW Magalhaes MA Yan M Crow MR. The axonal repellent, Slit2, inhibits directional migration of circulating neutrophils. J Leukoc Biol (2009) 86(6):1403–15. doi: 10.1189/jlb.0609391 19759280

[164] Chaturvedi S Yuen DA Bajwa A Huang YW Sokollik C Huang L. Slit2 prevents neutrophil recruitment and renal ischemia-reperfusion injury. J Am Soc Nephrol (2013) 24(8):1274–87. doi: 10.1681/ASN.2012090890 PMC3736708 23766538

[165] Prasad A Qamri Z Wu J Ganju RK. Slit-2/Robo-1 modulates the CXCL12/CXCR4-induced chemotaxis of T cells. J Leukoc Biol (2007) 82(3):465–76. doi: 10.1189/jlb.1106678 PMC2286829 17565045

